# A factorial randomised controlled trial to identify efficacious self-regulation techniques in an e- and m-health intervention to target an active lifestyle: study protocol

**DOI:** 10.1186/s13063-019-3456-7

**Published:** 2019-06-10

**Authors:** Helene Schroé, Celien Van der Mispel, Ilse De Bourdeaudhuij, Maïté Verloigne, Louise Poppe, Geert Crombez

**Affiliations:** 1 Ghent Health Psychology Lab, Department of Experimental-Clinical and Health Psychology, Faculty of Psychology and Educational Sciences, Ghent University, Henri Dunantlaan 2, Ghent, 9000 Belgium; 2 Research Group Physical Activity and Health, Department of Movement and Sports Sciences, Faculty of Medicine and Health, Ghent University, Watersportlaan 2, Ghent, 9000 Belgium

**Keywords:** EHealth, mHealth, Self-regulation, Behaviour-change techniques, Study protocol, Physical activity, Sedentary behaviour

## Abstract

**Background:**

Sufficient physical activity and a limited amount of sedentary behaviour can prevent a range of chronic diseases. However, most adults do not meet the recommendations for physical activity and sedentary behaviour. Effective and engaging interventions are needed to change people’s behaviour. E- and m-health interventions are promising, but unfortunately they result in small effects and suffer from high attrition rates. Improvements to intervention content and design are required. Qualitative research has revealed the need for clear and concise interventions. Furthermore, many interventions use a range of behaviour-change techniques, and it is yet unknown whether these techniques are equally important to obtain behaviour change. It may well be that a limited set of these techniques is sufficient. In this study, the aim is to experimentally investigate the efficacy of three behaviour-change techniques (i.e. action planning, coping planning and self-monitoring) on physical activity, sedentary behaviour and related determinants among adults.

**Methods:**

In a 2 x 2 x 2 factorial trial participants will be randomly allocated to eight groups (including one control group). Each group will receive a different version of the self-regulation-based e- and m-health intervention ‘MyPlan 2.0’, in which three behaviour-change techniques (i.e. action planning, coping planning, self-monitoring) will be combined in order to achieve self-formulated goals about physical activity or sedentary behaviour. Goal attainment, and levels of physical activity and sedentary behaviour will be measured via self-report questionnaires.

**Discussion:**

This study should provide insight into the role of various behaviour-change techniques in changing health behaviour and its determinants. Its experimental and longitudinal design, with repeated measures of several determinants of behaviour change, allows an in-depth analysis of the processes underlying behaviour change, enabling the authors to provide guidance for the development of future e- and m-health interventions.

**Trial registration:**

This study is registered as MyPlan 2.0 as a clinical trial (ID number: NCT03274271). Release date: 20 October 2017.

**Electronic supplementary material:**

The online version of this article (10.1186/s13063-019-3456-7) contains supplementary material, which is available to authorized users.

## Background

Sufficient physical activity (PA) and limited sedentary behaviour (SB) may reduce adverse health outcomes such as cardiovascular diseases, diabetes type 2, depression and cancer [1–7]. Physical activity is defined as ‘any bodily movement produced by skeletal muscles that requires energy expenditure’ [8]. The level of energy expenditure is expressed in metabolic equivalents (METS). Three levels of PA are defined based on these METs; ‘light-intensity physical activities’ such as doing the dishes or standing at your desk, ‘moderate-intensity physical activities’ such as cleaning windows or cycling to work and ‘vigorous-intensity physical activities’ such as running or chopping wood [9]. Given the examples above, PA means more than ‘exercise’ alone. For adults, it is recommended to be physically active at moderate to vigorous intensity (MVPA) for 30 min each day [10]. However, 31.1% of the worldwide population is physically inactive, with even higher levels of inactivity in higher-income countries [11]. Sedentary behaviour is defined as ‘any waking behaviour characterised by an energy expenditure ≤ 1.5 METs while in a sitting, reclining or lying posture’ [12]. For example, activities such as working at the computer while sitting, watching television or driving a car are considered SBs; however, sleeping is not. For SB, only a few countries have developed recommendations. In Belgium, for example, the ‘Flemish Institute for a Healthy Living’ (Vlaams Instituut Gezond Leven) recommends that adults limit prolonged periods of sitting and interrupt sitting time every 20–30 min [13]. Nevertheless, adults in Belgium sit on average 8.3 h per day [14]. Across Europe, adults report more than 5 h of sitting per day, with 18.5% sitting more than 7.5 h per day [15]. Consequently, there is a strong need for effective interventions that promote PA and limit SB on a large scale.

E- (electronic) and m- (mobile) health is defined as ‘the use of information and communications technology, especially the Internet, to improve or enable health and health care’ [16]. E- and m-health interventions have the potential to reach large groups in an interactive way [17, 18]. Furthermore, they are effective in changing health behaviours such as PA and SB [19–22]. Although these interventions are promising, effect sizes are often small and high attrition rates (60–80%) are observed [21, 23]. There is a need to improve e- and m-health interventions and to engage users for a sufficiently long time in order to induce long-term behavioural change.

To improve users’ engagement, interventions may be tailored to the specific needs of the users and only contain personally relevant information [24, 25]. Furthermore, e- and m-health intervention sessions should be clear and concise [26]. It is important that interventions offer effective and sufficient content in order to establish behaviour change. Informed by theoretical models of behaviour change (for example: the Health Action Process Approach (HAPA) [27]), interventions often include several behaviour-change techniques such as action planning, coping planning, prompting self-monitoring of behaviour, etc. [28]. The effectiveness of these techniques is well-established [29–31], but including various techniques increases the risk of making the sessions time-consuming and less engaging for the users. Up to now, it is unknown whether these techniques are equally important to obtain behaviour change, or whether a limited set of these techniques is sufficient. Gaining insight into the efficacy of different behaviour-change techniques can provide guidance in removing particular behaviour-change techniques in order to create more efficient interventions [32]. Therefore, studies using an experimental approach are greatly needed [33–36]. The publication of research protocols is essential to avoid publication bias and outcome reporting bias [37]. In this paper we report our research protocol taking into account the Standard Protocol Items: Recommendations for Interventional Trials (SPIRIT) guidelines [38]. The completed SPIRIT Checklist has been included as Additional file 4 and the SPIRIT Figure can be found in Fig. 3.

## Aims and objectives

Our primary aim is to investigate which combination of behaviour-change techniques of an e- and m-health intervention in adults results in an optimal efficacy. We will explore the role of three behaviour-change techniques (i.e. action planning, coping planning and self-monitoring) on PA, SB and the attainment of self-formulated goals. We will apply an experimental approach and conduct a 2 x 2 x 2 factorial randomised trial using ‘MyPlan 2.0’, an e- and m-health intervention to increase PA and to reduce SB among adults.

Our second aim is to investigate the effect of the self-regulation techniques on mediating change-related determinants (i.e. self-efficacy, motivation, intention, action planning, coping planning, self-monitoring, risk perception and outcome expectancies) and to examine possible moderating effects of demographic variables. Hypotheses about mediation and moderation are informed by the HAPA [27, 39, 40], a self-regulation model that includes both pre-intentional as well as post-intentional determinants of behaviour change.

Our third aim is to investigate whether our intervention is effective for particular groups of participants. According to the HAPA model, individuals can be divided into three groups, based on their ‘mindset’ regarding behaviour change. The first group or ‘pre-intenders’ consists of individuals that have not yet formed an intention. The second group or ‘intenders’ have formed an intention to change their behaviour, but they do not yet perform the action itself regularly. The third group or ‘actors’ already perform the action for which they previously formed an intention. Interventions can be tailored upon these different mindsets, hence targeting mostly pre-intentional or post-intentional determinants. Because our intervention focusses on bridging the intention-behaviour gap, we expect that our intervention works better for intenders than for pre-intenders and for actors.

## Methods

### Study design

The design of the study is a 2 x 2 x 2 factorial randomised trial, in which participants are randomly allocated to eight different groups (including one control group). Each group will receive a different version of the e- and m-health intervention, in which three different behaviour-change techniques (i.e. action planning, coping planning, self-monitoring) are combined. The control group will receive only tailored feedback, information and tips and tricks. Table 1 shows the combination of the techniques within each of the groups.

**Table 1:** Behaviour-change techniques provided for each group

	Action planning	Coping planning	Self-monitoring
Group 1	+	+	+
Group 2	+	+	–
Group 3	+	–	+
Group 4	–	+	+
Group 5	+	–	–
Group 6	–	+	–
Group 7	–	–	+
Group 8 (control group)	–	–	–

### Intervention

Each group will receive a different version of the e- and m-health intervention MyPlan 2.0. MyPlan 2.0 is based upon a previous e-health intervention, called ‘MyPlan 1.0’, which was systematically developed using the intervention mapping protocol [41]. MyPlan 1.0 is a web-based intervention to promote PA and fruit and vegetable intake in the general population, and has been tested in general practice. The programme has shown to be effective in improving healthy behaviour [42–44]. However, high attrition rates were observed and researchers experienced problems with the implementation by general practitioners [42–44]. Therefore, the programme has been further developed into MyPlan 2.0, which participants can use autonomously at home. In order to increase engagement, different adaptations were made based on quantitative and qualitative research [24, 26, 45–47]. MyPlan 2.0 consists of a website (www.mijnactieplan.be/meerbewegen) as well as a mobile application (app). It aims to increase PA and decrease SB in the general adult population (MyPlan 2.0 is also used for another independent study in our research group, which focusses on adults with diabetes mellitus type 2 [48]). The website consists of five weekly sessions, whereas the app offers daily support. Similar to MyPlan 1.0, MyPlan 2.0 is based on self-regulation theory [49, 50] and includes various self-regulation-based behaviour-change techniques [28, 51] in order to bridge the intention-behaviour gap [27, 52, 53]. In both the website and the app, the techniques can easily be removed or added in order to create the different research conditions.

Before the start of the intervention, participants choose which behaviour they want to improve. Participants are free to select either PA or SB. The structure of MyPlan 2.0 is the same for both PA and SB. After behaviour selection, the allocation to one of the research groups takes place. After being allocated into a research group, users will receive different website links to the corresponding interventions.

#### The website

**Fig. 1:**
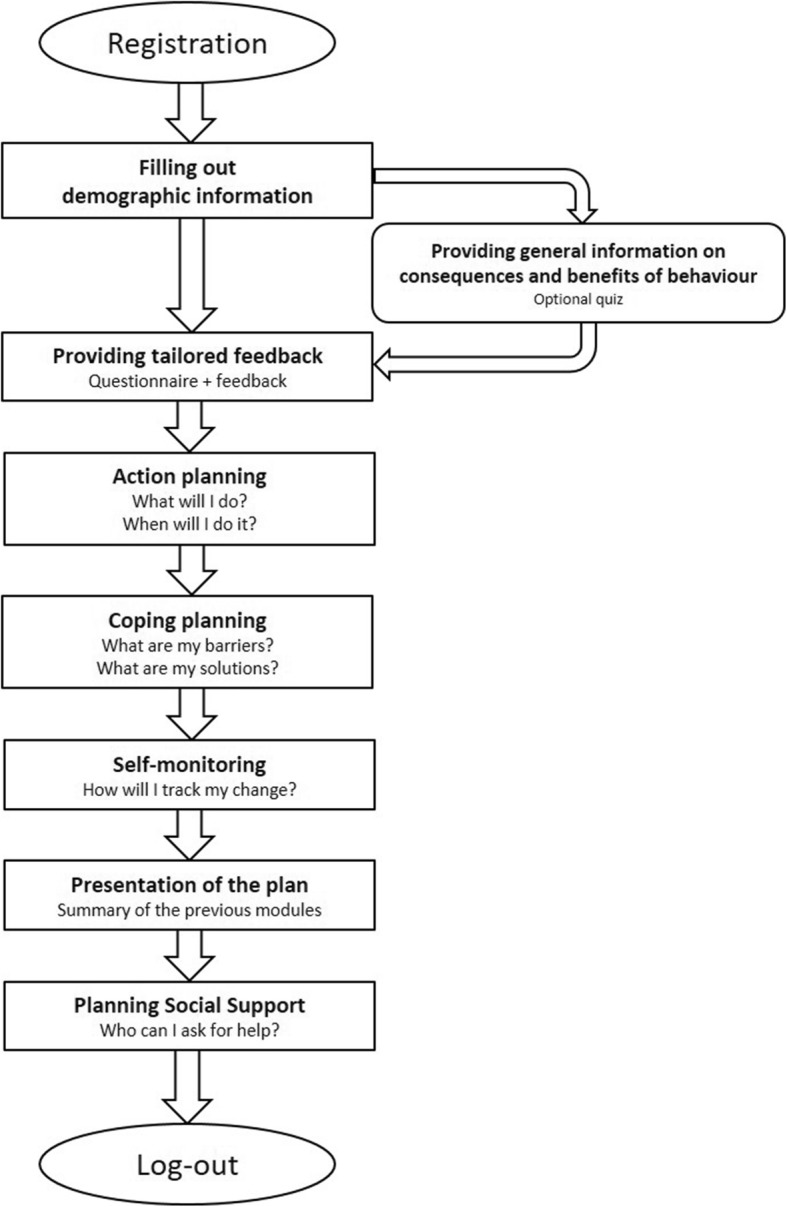
Flow of the first session on the website

**Fig. 2:**
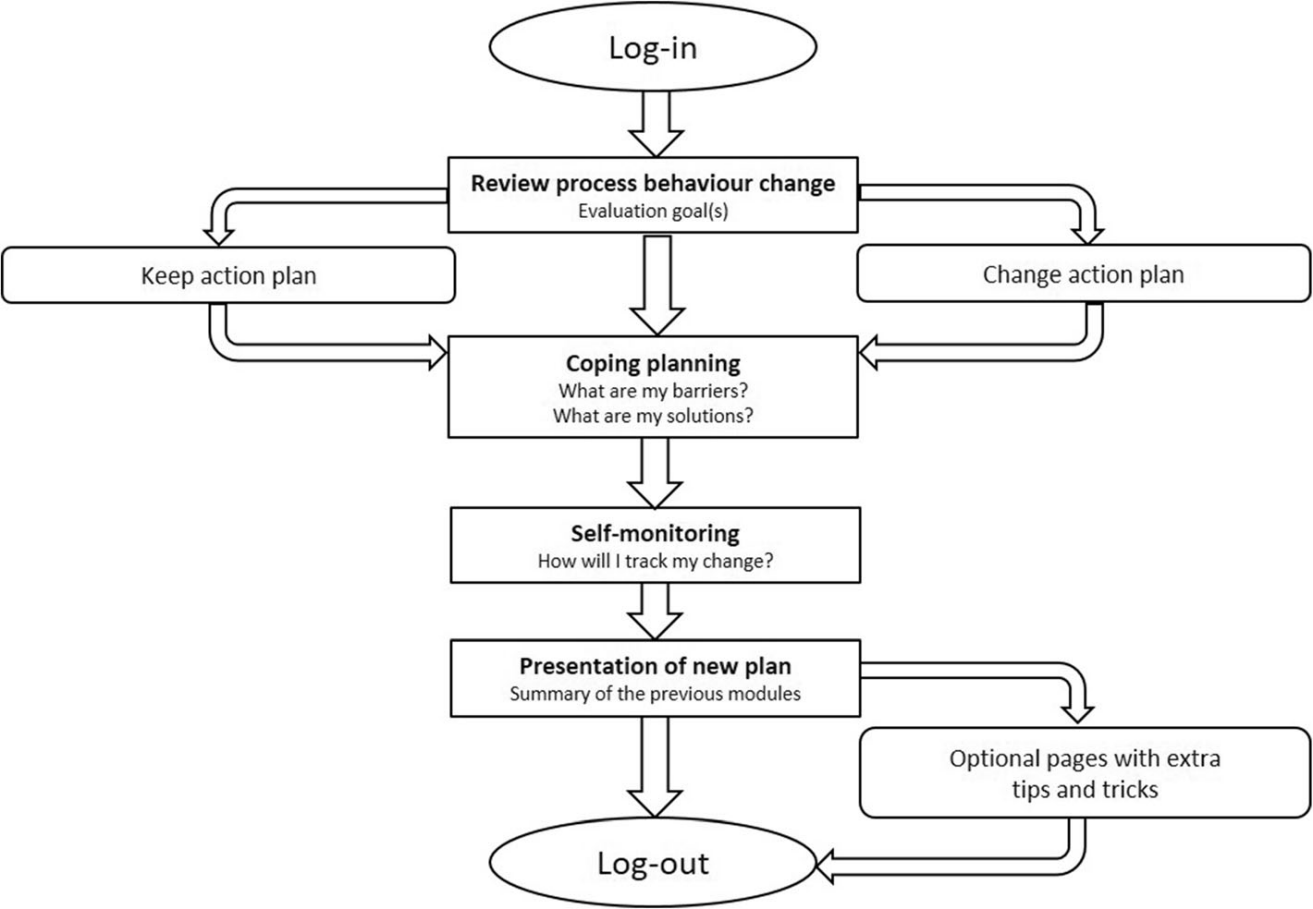
Flow of follow-up sessions 2, 3, 4 and 5 on the website

The flow of the website is depicted in Figs. 1 and 2. In the first website session, users start with a short registration procedure, in which they have to register with their email address. They also have to fill in some demographic information, such as name, age and sex. Thereafter, they are offered the possibility to take a quiz. Via quiz-like questions, participants are provided with general information on the consequences and benefits of the selected behaviour (see Additional file 1). Then, all participants have to fill out either the short version of the ‘International Physical Activity Questionnaire’ (IPAQ) or the Last 7-days Sedentary Behaviour Questionnaire (SIT-Q-7d) (based upon their chosen behaviour) [54, 55]. Based on the answers in this questionnaire, users receive tailored feedback: their current level is compared to the health guidelines and suggestions are made on how they can improve their behaviour (e.g. in which domains). In order to guide them through actual behaviour change, users receive various behaviour-change components, according to the research condition they are assigned to.

Users who receive *the action planning component* can start making their own plan for behaviour change by specifically defining what they are going to do, when and where. Various examples are provided and success stories of other users are shown as well.

*The coping planning component* is implemented by letting users choose which barriers they believe they will experience and providing a list of possible solutions for each of these barriers. Users can also provide other barriers and suggest their own solutions. Examples of how to create good coping plans are given.

In *the self-monitoring component*, users are prompted to self-monitor their goal. They are prompted to do this via the app, but they can also select other options (such as writing it down in their diary or on their calendar). A calendar page on which they can easily indicate their progress can be downloaded from the website.

Finally, a summary is shown of the action plan, and/or coping plan, and/or the plan to monitor this. Note that the content summary will vary as a function of the experimental condition. Users get the possibility to print this summary. Next, they can read about how social support can be elicited (e.g. talking to their partner, informing colleagues of their plan, using social platforms …).

In the subsequent four sessions on the website, users reflect on the process of behaviour change during the past week. They can maintain or adapt the action plan, coping plan and/or their self-monitoring method. Each week, extra tips and tricks to enhance behaviour change are added to the intervention content in order to optimally engage and support the users. Screenshots of the website can be found in Additional file 1.

#### The mobile application

The app of MyPlan 2.0 is synchronised with the website and provides user support on a daily basis. The included self-regulation techniques differ as a function of the experimental group (the app system recognises the email address and the user will get the app version of the group they are assigned to). *The action planning component* is implemented in the app by providing users with the option to change their plan throughout the week. Furthermore, the app reminds users of their plan by sending a message on the scheduled moment. For example, if a user planned to go for a walk on Tuesday, they receive a notification on Tuesday morning as a reminder. In *the coping planning component*, users can select barriers and receive an overview of possible solutions. If users are assigned to *the self-monitoring component*, a notification is sent at the end of each day, in which users are asked to what extent they succeeded in their plan (i.e. a score ranging from 1 ‘not succeeded’ to 5 ‘very well succeeded’). The scores are displayed in a graph and can be accessed in the self-monitoring module. If a user indicates a score of less than 3, they receive the advice to go to the coping planning component. Additionally, the app aims to engage users via elements of gamification (i.e. ‘the use of game design elements in non-gaming contexts’ [56], p.9) such as quizzes and collecting medals for completing website sessions or self-monitoring measures. Screenshots of the application can be found in Additional file 2.

### Participants

This study targets adults in the general Belgian population. The following inclusion criteria will be taken into account: (1) being ≥ 18 years old, (2) Dutch-speaking, (3) having Internet access and (4) being the owner of a smartphone. Before entering the study, people have to complete the ‘Physical Activity Readiness Questionnaire’ (PAR-Q) [57] which is a screening instrument to detect individuals at risk for adverse effects when being more physically active. The PAR-Q is a self-report instrument consisting of seven items to be answered by ‘yes’ or ‘no’. Participants will be excluded when one item or more will be endorsed. Participants receiving concomitant care or other interventions will not be excluded.

A convenience sample will be attained by recruiting possible participants in the city library of Ghent (i.e. ‘De Krook’), in city halls and shopping malls. Also participants will be recruited by social media, contacting participants of previous studies who indicated as having interest in other research, recruiting acquaintances and students, and applying snowball sampling.

Based on the meta-analysis of Conn et al. (2011) [58], we would like to be able to observe a small effect size of 0.19. To detect within- and between-subjects effects under significance level alpha = 0.05, and with a power of 0.80, a sample of 260 participants is needed. However, we will aim to recruit approximately 480 participants to take attrition into account. As such, an attrition rate of 46% of the participants is acceptable within this study, which seams feasible to the researchers. This is because actions to decrease drop-out were taken; no extensive questionnaires were used in MyPlan 2.0 in contrast to previous research (e.g. MyPlan 1.0) and, furthermore, the programme was adapted to increase user engagement based on both qualitative and quantitative research [46, 47].

The participants will be randomly allocated to eight equal groups (60 participants within one group). The randomisation will be done by the author of this study. Before recruitment of participants, blocked randomisation will be used to form an allocation list for the eight different groups. A computer random number generator (http://www.randomization.com) [59] with a block size of 40 will be used to assign the group numbers in random order within each block. The allocation list will be concealed for the researcher during the recruitment of participants. Each time a participant completes a pre-test measurement (at the library, city hall, shopping mall or at home; see Fig. 4) an automatic email will be sent to the researcher. After receiving this email, the participant will be allocated to the next number on the allocation list and will consequently be assigned to a group. By doing this, the allocation will be concealed until participants are enrolled and assigned to the intervention [60]. After assignment, the researcher will not be blind for the allocated intervention: the researcher will send participants different website links to their corresponding intervention. The participants on the other hand will not be aware of the different conditions of the study: they will only receive one website link (corresponding with their intervention).

### Measurements

**Fig. 3:**
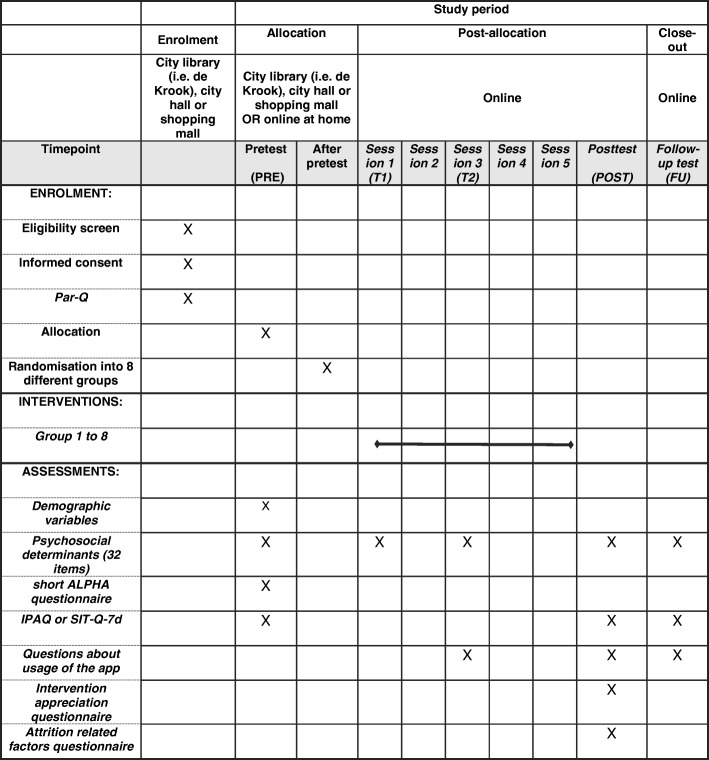
Standard Protocol Items: Recommendations for Interventional Trials (SPIRIT) Figure. The figure shows the phases of the trial and the overview of variables measured at five time points

To answer our research questions, outcomes will be measured at five time points: 1 week before the intervention (PRE), immediately after session 1 (T1), immediately after session 3 (T2), maximum 1 week after the intervention (POST) and 1 month after the intervention (FU). Figure 3 shows which variables will be measured at each time point.

PA and SB, the primary outcomes, will be assessed via self-report measures. The IPAQ [61] will be used to measure self-reported total PA and moderate-to-vigorous PA across different domains (i.e. work, home, leisure time, transport). A 7-day sedentary behaviour self-report questionnaire (SIT-Q-7d) [62] will be used to measure domain-specific total sedentary time in five domains (meals, transportation, occupation, non-occupational screen time and other sedentary time). Both questionnaires have shown fair to good reliability and validity [61, 62]. Furthermore, users will report weekly to what extent they achieved their PA or SB goal (secondary outcome measure). After seeing the goal(s) they set the week before, the following question will be asked: ‘Did you reached your goal(s)?’: ‘Yes’, ‘No’ or ‘A little’.

To evaluate our second aim of the intervention, psychosocial determinants of behaviour (i.e. self-efficacy, motivation, outcome expectancies, risk perception, intention and the degree to which action planning, coping planning, self-monitoring is used in daily life) will be measured via a set of 32 items (i.e. at least three items per determinant). This set was iteratively developed and validated by an expert panel, using (1) cognitive interviewing [63, 64] and (2) a procedure for content validity which was based on the method of Johnston et al. (2014) [65]. In a first step a large pool of items were created measuring the personal determinants based upon the HAPA model [27]. Next, 11 experts in the field of self-regulation theory (which the determinants were based on) rated to what extent each item measured each construct of the HAPA model. Also feedback on the formulation was provided, and some items were reworded. Finally, a team of three researchers selected the best scoring items.

Participants will also complete questions regarding demographic information (age, sex, educational level, occupation, marital status, height and weight) and intervention usage (see ‘Process evaluation’). Furthermore, the short version of the ‘Instruments for Assessing Levels of Physical Activity and fitness’ (ALPHA) questionnaire [66] will be administered, which measures the perceptions of the physical environment (e.g. Are there many shops within easy walking distance of your home?). This questionnaire shows good reliability and predictive validity [66].

### Process evaluation

To gain insight in the different aspects of intervention integrity, we will conduct a process evaluation. Five components will be evaluated, based on the guidelines provided by Saunders, Evans and Joshi [67]: fidelity, dose delivered, dose received, recruitment and reach, and context.

#### Fidelity

Fidelity, also defined as quality, refers to the degree in which the intervention was carried out as planned. This will be assessed by rating the quality of the action and coping plans that participants create via the website and app. This will be done by scoring the plans both on instrumentality and specificity [68, 69].

#### Dose delivered

Dose delivered, or completeness, is the quantification of components of the website that were actually delivered in comparison to the components available. This will be measured by keeping track of the users’ website and app log data and counting how many online sessions they initiated and completed. We will keep track of how long users spend time on the website and app using the different components, how long it takes them to log back in and complete the intervention, how many times they have to be reminded for logging in again, if users complete optional components (such as the quizzes), and how many times they adapt their plan. Furthermore, at three different points in time (after session 3, post-intervention and follow-up intervention) users will rate how often they use the app (i.e. never, less than 1 day/week, 1 to 2 days/week, 3 to 4 days/week, 5 to 6 days/week, daily).

#### Dose received

Dose received consists of two parts: exposure (i.e. the quantification of components that were actually received) and satisfaction (i.e. how users perceive the intervention). Because the intervention is directly delivered to the participants, exposure to the intervention will be identical to ‘dose delivered’. Consequently, log data of the website and app can be consulted. In order to gain insight into the satisfaction of users with the intervention, a questionnaire regarding appreciation of the intervention and attrition-related factors will be administered at the end of the intervention. The questionnaire about appreciation is based on items from two other questionnaires assessing experiences with e-health interventions [70, 71] and can be found in Table 2. The attrition-related factors will be assessed using questions based on the factors described in an article of Eysenbach [23]. The question about taking part in other programmes targeting a healthy lifestyle is also measured at the follow-up measurement. These questions can be found in Table 3. Furthermore, actual attrition rates will be described and reasons for attrition will be examined. During the study, participants who do not log in for the online sessions, will be contacted and information about the described problems with the website and the app will be kept in a record. At the end of the intervention, the following question will be asked to all users: ‘Did you experience any problems using the mobile application?’. Three response options will be provided: (1) Yes, I could not use the app, (2) Yes, but I could use the app from time to time (3) No, I could use the app without problems. Moreover, participants who discontinue using the intervention will be asked to provide the reasons for dropping out during a telephone call. First they will be asked to freely describe why they stopped using the intervention, afterwards they will be asked to rate possible reasons (i.e. loss of interest in the matter, lack of time, achievement of health norms, loss of motivation for behaviour change, lack of relevant content in the intervention, technical problems and medical or emotional problems) on personal relevance.

**Table 2:** Overview of the questions about the appreciation of the website and mobile application

Question	Scale
Appreciation of the website	
Overall, how do you rate the website of ‘MyPlan 2.0’?	1 (very poor) – 10 (outstanding)
How do you rate the quiz?	1–10
How do you rate the questionnaire and the accompanying feedback?	1–10
How do you rate the action planning module?	1–10
How do you rate the coping planning module?	1–10
How do you rate the tips and tricks section?	1–10
How do you rate the feedback in the follow-up sessions?	1–10
Appreciation of the mobile application	
Overall, how do you rate the mobile application of ‘MyPlan 2.0’?	1–10
How do you rate the quizzes?	1–10
How do you rate the monitoring module?	1–10
How do you rate the action planning module?	1–10
How do you rate the coping planning module?	1–10
How do you rate the points collection module?	1–10
Appreciation of ‘MyPlan 2.0’ as a whole	
Was the information and support delivered by ‘MyPlan 2.0’ understandable?	1 (not at all) – 5 (very much)
Was the information and support delivered by ‘MyPlan 2.0’ useful?	1–5
Was the information and support delivered by ‘MyPlan 2.0’ of personal relevance for you?	1–5
Was the information and support delivered by ‘MyPlan 2.0’ motivating?	1–5
Did you enjoy using ‘MyPlan 2.0’?	1–5

**Table 3:** Overview of the attrition-related factors from the participants

Question	Scale
‘MyPlan 2.0’ lived up to my expectations	1 (not at all) – 5 (very much)
The website of ‘MyPlan 2.0’ is user friendly	1–5
The mobile application of ‘MyPlan 2.0’ is user friendly	1–5
My GP reacted positively regarding my participation in ‘MyPlan 2.0’	1–5
My friends and family reacted positively about my participation in ‘MyPlan 2.0’	1–5
‘MyPlan 2.0’ helped me to be more physically active/to sit less	1–5
The personal contact with the researchers of ‘MyPlan 2.0’ was an additional reason for me to participate	1–5
Going through ‘MyPlan 2.0’ took a lot of my time	1–5
Filling out the questionnaires took a lot of my time	1–5
^a^I also took part in other programmes targeting my healthy lifestyle (e g. consulting a dietitian, starting to run, …) ^a^Which ones?	Yes/No
While taking part in the study drastic changes in my life occurred (e.g. death of a family member, had a (grand)child, new job, etc.)	1–5
I can work well with a computer	1–5
When I have computer problems, I can rely on others to help me	1–5
I had doubts about participating in this study	1–5

^a^This question is also measured at the follow-up measurement

#### Recruitment and reach

Recruitment and reach refer to the proportion of the target population that participated in the intervention and how the sample was recruited, respectively. Because recruitment of participants will be done via different channels, we will keep track of how many participants were recruited via each channel. This can provide us with insights regarding where and how interventions can be implemented or disseminated.

#### Context

Context discusses possible influences of the environment on intervention implementation and outcomes. The most important influence of context on the implementation of the intervention will be assessed via the short version of the ALPHA questionnaire [66], as mentioned above, and the following question: ‘Did physical or emotional problems prevent you from being more physically active/less sedentary?’

### Data collection procedures

**Fig. 4:**
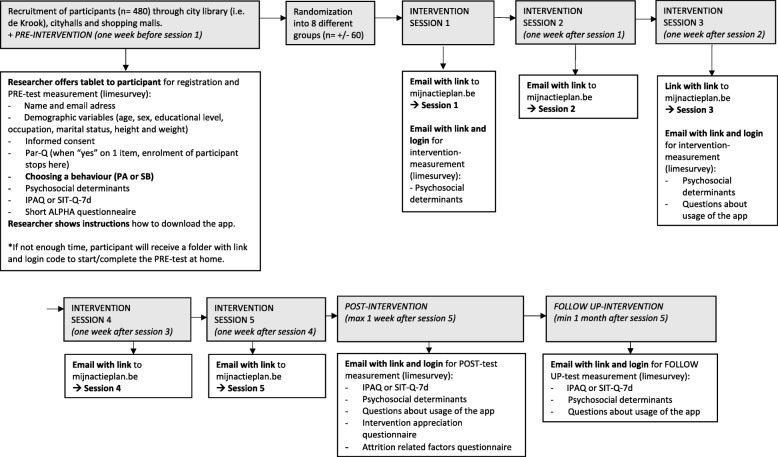
Flowchart of data collection

The flowchart of the data collection procedure can be found in Fig. 4. Data collection started in March 2018 and is aimed to finish in April 2019. Participants will be recruited in the city library of Ghent (i.e. ‘De Krook’), in city halls and shopping malls. If a participant is willing to co-operate in the study, they will be offered a tablet and the pre-test measurement will take place. The participant will provide informed consent, choose a behaviour (PA or SB) and complete the questionnaires (see Fig. 3) via an online survey tool (https://www.limesurvey.org/) [72]. Subsequently, researchers will provide instructions on how to download the app. If the participant does not have sufficient time to fill out the questionnaire, they will receive a folder with a link and login code to start/complete the pre-test measurement at home. After completion of the pre-test measurement, the participant will be allocated to one of the eight groups, and will receive an email with the web-link to the appropriate version of the intervention. MyPlan 2.0 and its different conditions can only be accessed via the link sent by the researcher and, later on, via the provided email address of the participant. Participants have no access to the other experimental conditions of MyPlan 2.0. One week after each intervention session, an automatic email to encourage people to start with the following session will be sent. If participants take longer than expected to a complete session, several actions will be taken. Participants who do not start or complete a session after 1 week will be sent an email reminder. When there is no reaction after 2 weeks, participants will receive a telephone call from the researcher. When there is no reaction after 3 weeks, the participant will be considered as a drop-out.

Maximum 1 week after completing the five online intervention sessions, participants will receive an email with the link to the post-intervention measures about PA, SB and determinants of behaviour change (see Fig. 3). The post-intervention measures will also include questions about app usage, intervention appreciation and attrition-related factors, necessary for the process evaluation. One month after intervention completion, participants will again receive an email with a link to the follow-up measures (see Fig. 3).

In order to be able to conduct mediation and moderator analyses and track the change in the determinants of behaviour change, participants will complete the item set regarding the determinants after session 1 and session 3. The link and login will be integrated in the email for sessions 1 and 3.

### Data management and statistical analysis

Data management and analyses will be monitored and supervised by the team of six researchers and a PhD guidance committee. The data management plan, which describes data storage, management and confidentiality, is provided in Additional file 3. Results of the study will be published anonymously in scientific journals and via public health communication channels. For all publications, the APA guidelines for authorship eligibility will be followed.

Statistical analysis will be performed after data collection is completed. The data of our factorial randomised controlled trial will be analysed using R version 3.2.5 [73].

Descriptive statistics will be provided for the total sample and for all groups. Baseline differences between all groups regarding demographic variables, determinants of behaviour change and the level of PA and SB will be explored by using one-way analysis of variance (ANOVA) (for the continuous variables) and chi-square tests (for the nominal variables). If baseline differences occur in terms of age and gender, they will be included as a covariate factor in the analyses. We will explore the effect of other baseline differences via sensitivity analyses.

To handle our missing data, missing data mechanisms will be investigated. First, we will check whether the missing values are missing completely at random (MCAR) or missing at random (MAR) using the Little Missing Completely At Random test in R [74]. If our data are MCAR or MAR, multiple imputation will be used which provides valid results when the data are at least missing at random [75]. The analysis will be conducted in R using the MICE package [76]. However, one cannot rule out the possibility that the data will be missing not at random (MNAR), therefore sensitivity analyses will also be conducted [75].

To evaluate the primary aim of the intervention, PA and SB will be analysed in one statistical analysis. The scores of PA and SB will be standardised (z-score) in order to obtain a common metric. For PA, the mean and SD of the population will be based on self-report IPAQ data self-report IPAQ data from Belgian adults (m_totalPA_ = 831.5 min/week, SD_totalPA_ = 579.8; m_MVPA_ = 459.9 min/week, SD_MVPA_ = 407.2 min/week) [77]. For SB, the mean and standard deviation of the population will be based on self-report SIT-Q-7d data from Belgian adults (m_totalSB_ = 9,57 h/day, SD_totalSB_ = 3,20 h/day) [78]. The z-scores for SB will be multiplied by − 1. That way the amount of healthy behaviour is reflected in positive scores. Mixed models (two levels: repeated measures clustered within participants) will be performed using the ‘lme4-package’ [79] to investigate the effect of the (combination) of the three behaviour-change techniques (i.e. action planning, coping planning and self-monitoring) on behaviour change [80]. The participants’ choice of target behaviour (i.e. PA or SB) will be included in the analysis as a moderator. Furthermore, to evaluate our secondary outcome, logistic regression analyses will be used to investigate whether the process on goal achievement has an influence on the primary outcome.

To evaluate the secondary aim of the intervention, PA and SB will be analysed both together for analysing the mediating effect of personal determinants. This because all participants filled out one version of the personal determinants (i.e. the version focussing on PA or the version focussing on SB). These outcome variables will be considered as personal determinants regarding the chosen health behaviour rather than personal determinants regarding increasing PA or decreasing SB. Mediating effects of the personal determinants of behaviour will be investigated using structural equation modelling [81]. Next, moderating effects of demographic variables will be identified via interaction terms by including them as an extra between factor in the analyses.

To evaluate the third aim of the intervention, three groups of participants will be identified based on their ‘mindset’ regarding behaviour change: ‘pre-intenders’, ‘intenders’ and ‘actors’. This will be based on their answer on the personal determinant ‘intention to change’. The moderating effect will be identified via interaction terms by also including them as an extra between factor in the analyses.

### Protection of privacy, ethical issues and expected adverse effects

The research protocol of this study was approved by the Committee of Medical Ethics of the Ghent University Hospital (Belgian registration number: B670201731996) and registered as a clinical trial at https://register.clinicaltrials.gov (ID number: NCT03274271). Participants will provide web-based informed consent regarding the study measures and log data of the website and app before enrolling in the study. They will be informed about the purpose and the design of the study, the potential benefits and risks, the voluntary basis of the study and how their personal information will be processed. Personal information will be coded and password-protected. Only researchers that are part of the research team will have access to the data. No incentive will be provided to the participants. It is expected that they will experience health benefits from an increase in PA or a decrease in SB. However, in the unlikely event that users may experience adverse effects, such as severe joint or muscle pain, participants will be encouraged to report adverse effects and will be advised to consult a physician in that case. Precautions to prevent adverse effects have been made by screening for health problems in the recruitment phase (see ‘Participants’).

## Discussion

There is a need for effective e- and m-health interventions that enhance behaviour change. Additionally, e- and m-health interventions need to be relevant, time-efficient and engaging. This study may contribute to a further understanding of how, why and for whom interventions work. By investigating the role of various behaviour-change techniques and determinants of behaviour change, we will be able to provide guidance for the development of future e- and m-health interventions. One of the strengths of this study is its experimental and longitudinal design, allowing us to isolate the effects of the separate behaviour-change techniques. To our knowledge, the influence of behaviour-change techniques has only been investigated as a combined effect or in a non-experimental way [53, 82, 83] and many authors in the field refer to the need for an experimental design [33–35]. Another strength of the study is the fact that not only PA and SB are measured via self-reports, but also a range of determinants is measured at different points in time, i.e. pre, post, at follow-up and during the intervention. This allows a thorough analysis of the processes of behaviour change that interventions can target. The results of such analysis can inform future interventions on how to tailor behaviour-change techniques to different participants in various phases of behaviour change. This study should provide a large and rich dataset to explore these research questions in detail. However, the study also has some limitations. First, PA and SB are measured by self-report questionnaires (IPAQ or SIT-Q-7d), which may lead to response and recall biases [84]. Future research may consider the use of objective measures of PA and SB. Second, to lower the burden for participants and thus preventing attrition, each determinant of behaviour change will be measured via only three items, which can compromise the reliability and validity of the measurement. However, the items that are included were iteratively constructed and validated by experts in health psychology. Third, the size of the groups will not allow us to draw conclusions about the three-way interactions. However, we are more interested in the main effects and the two-way interaction effects. Fourth, because participants can freely choose either to improve their PA or to reduce their SB, we cannot ensure that both versions of the programme will have the same number of participants. Lastly, due to attrition, it is possible that the sample size between groups will differ. Therefore, it will be important to focus on keeping the participants in the study via the use of email reminders and telephone calls.

## Trial status

The final website and app have been developed. The factorial randomised controlled trial started in March 2018 and we aim to finish in April 2019 as participants can start the study at different moments. The authors will report important protocol modifications at clinicaltrials.gov.

## Additional files

Additional file 1: Screenshots of the website. (PDF 1030 kb)

Additional file 2: Screenshots of the mobile application. (PDF 482 kb)

Additional file 3: Data management plan. (PDF 486 kb)

Additional file 4: Completed Standard Protocol Items: Recommendations for Interventional Trials (SPIRIT) Checklist. (PDF 172 kb)

## Data Availability

Not applicable.

## Abbreviations

ALPHA: Instruments for Assessing Levels of Physical Activity and fitness
FU: Follow-up, 1 month after the intervention
IPAQ: International Physical Activity Questionnaire
MVPA: Moderate to vigorous physical activity
PA: Physical activity
PAR-Q: Physical Activity Readiness Questionnaire
POST: Post-test, after the intervention
PRE: Pre-test, 1 week before the intervention
SB: Sedentary behaviour
SIT-Q-7d: Last 7-days Sedentary Behaviour Questionnaire
T1: Time point 1, after session 1
T2: Time point 2, after session 3
