# Supplementary material for: A factorial randomised controlled trial to identify efficacious self-regulation techniques in an e- and m-health intervention to target an active lifestyle: study protocol
Source: Trials. 2019 Jun 10;20:340. doi: 10.1186/s13063-019-3456-7 (PMC6558816; doi:10.1186/s13063-019-3456-7)
Supplement: Supplementary file 1 — Screenshots of the website. (PDF 1030 kb) [file 13063_2019_3456_MOESM1_ESM.pdf]

Myplan 2.0. Screenshots of the website

Figure 1a. Defining an action in the action planning module: “how will I do it?”

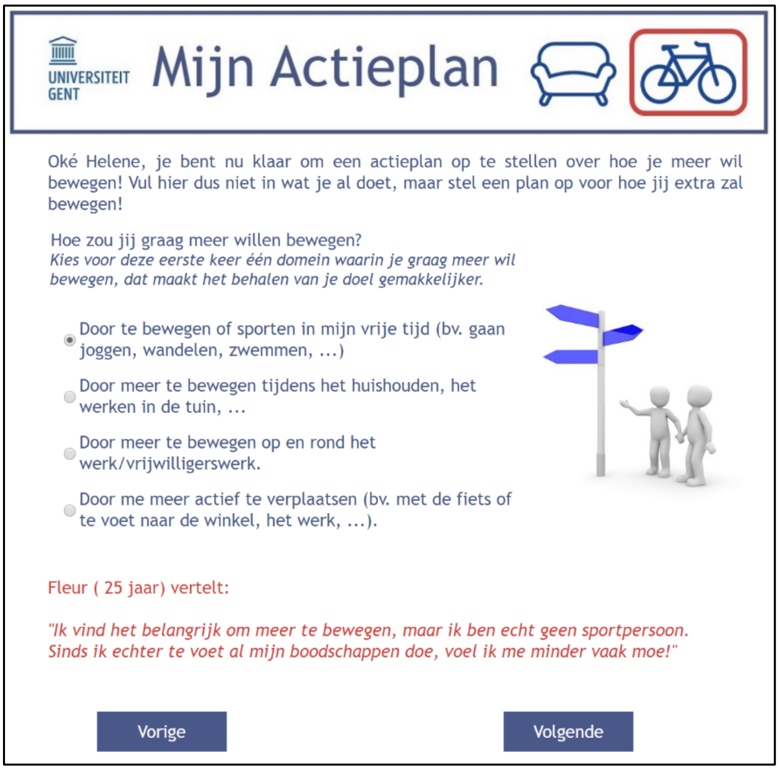


The text in red is a succes story.

Figure 1b. Defining an action in the action planning module: “What will I do?” “When will I do it?”

“Where will I do it?”

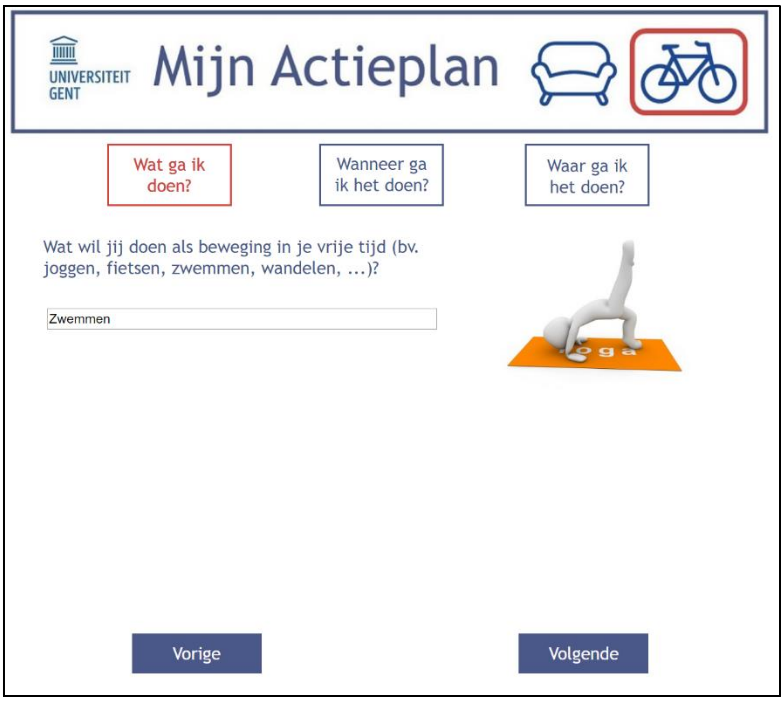

Figure 1c. The coping planning module: potential hindrances.

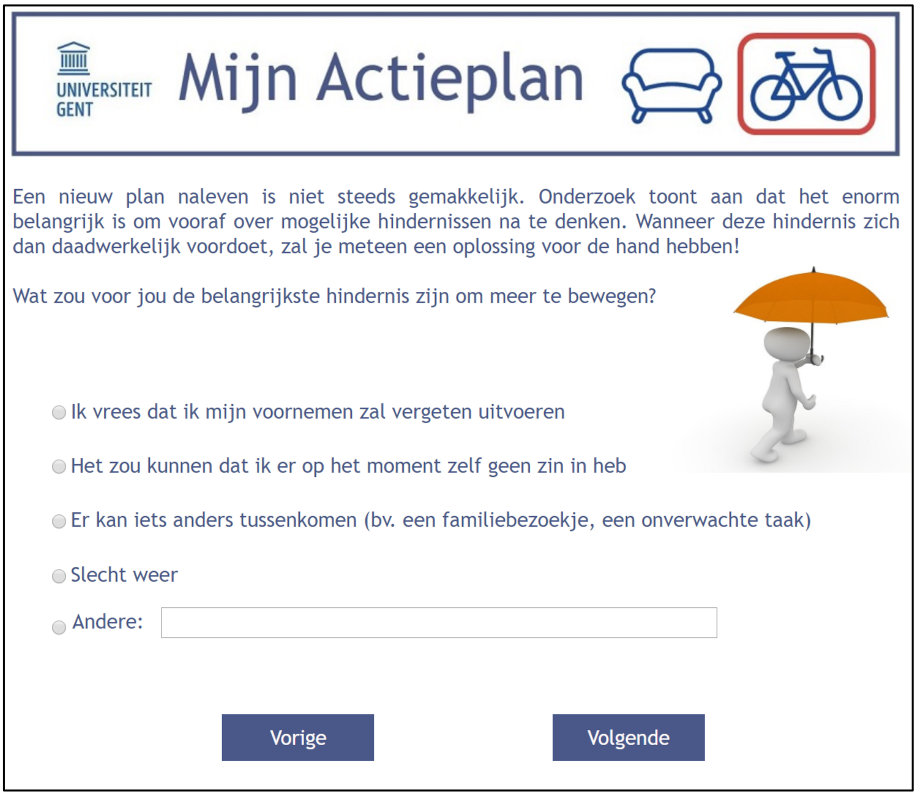


Translation: "Living up to a new plan is not always easy. Research shows that it is really important to consider potential hindrances in advance. When this hindrance appears, you will have an immediate solution at hand!"

Figure 1d. The coping planning module: possible solutions for hindrances.

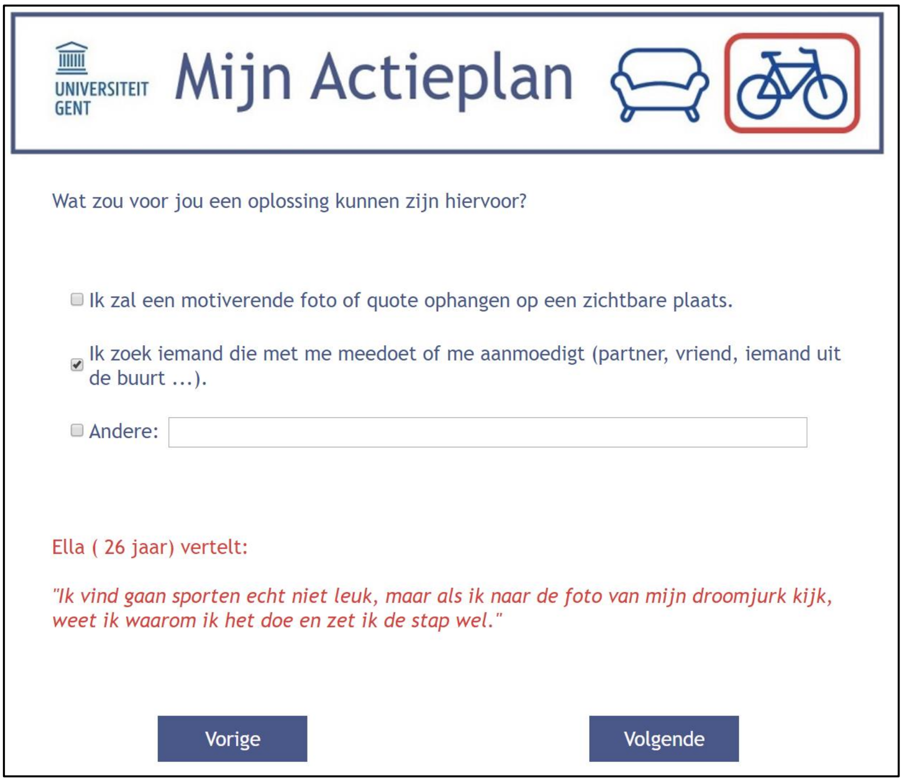


The text in red is a succes story.

Figure 1e. Example of a quiz question

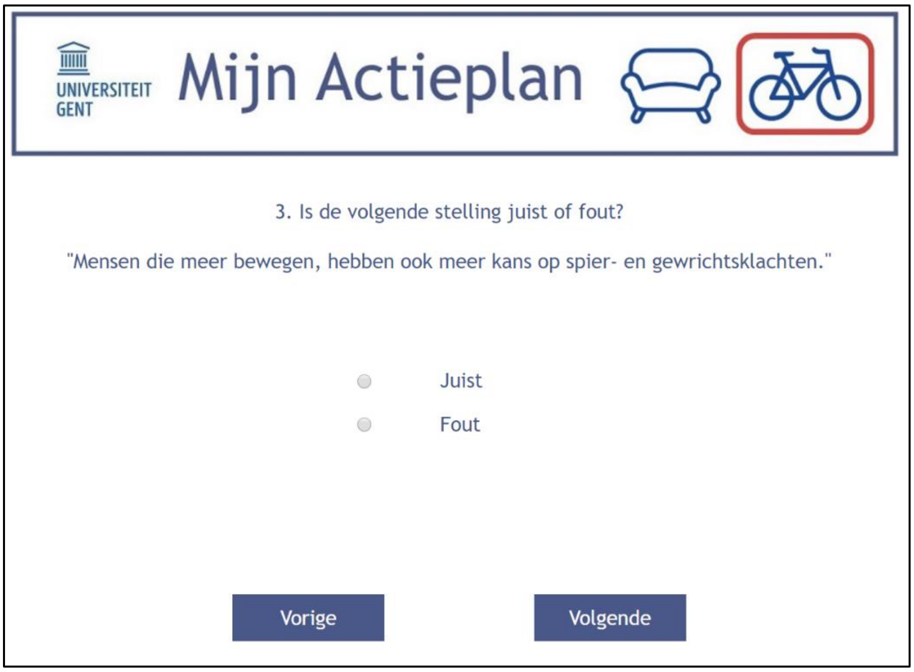


Translation: “Is the following statement true or false? If you move more, you will have more chance to develop muscle and joint pain”

Figure 2f. Solution and information of the quiz question

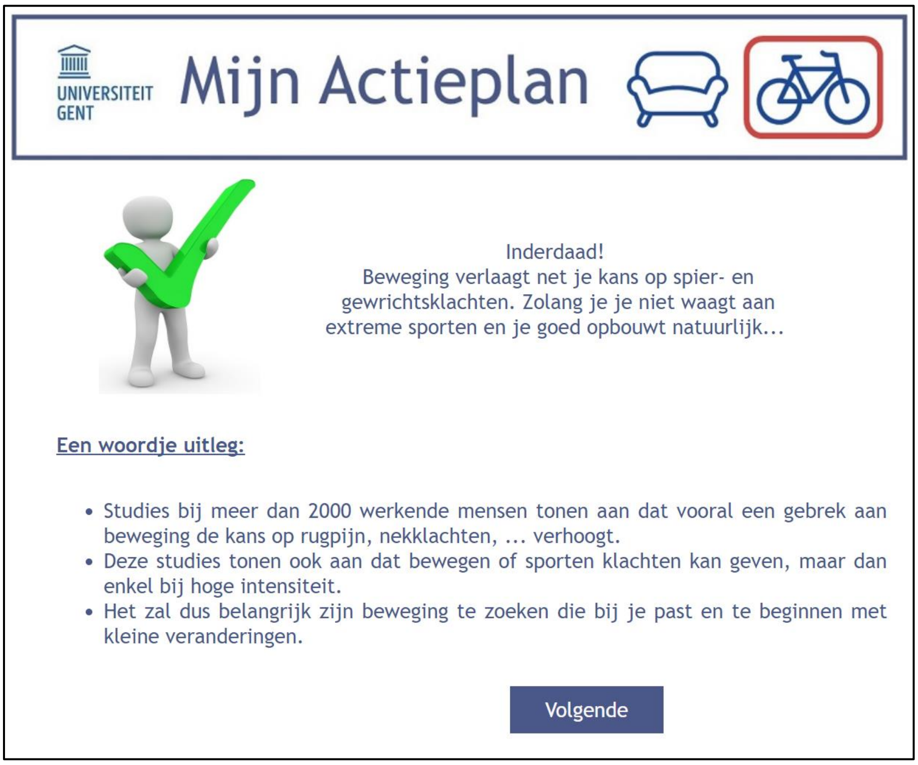


Translation: “Your answer was correct! More movement will reduce your chance on muscle and joint pain”
