# Supplementary material for: A factorial randomised controlled trial to identify efficacious self-regulation techniques in an e- and m-health intervention to target an active lifestyle: study protocol
Source: Trials. 2019 Jun 10;20:340. doi: 10.1186/s13063-019-3456-7 (PMC6558816; doi:10.1186/s13063-019-3456-7)
Supplement: Supplementary file 2 — Screenshots of the mobile application. (PDF 482 kb) [file 13063_2019_3456_MOESM2_ESM.pdf]

<u>Myplan 2.0. Screenshots of the mobile application</u>

Figure 1a. Action planning module: revising and adapting plans

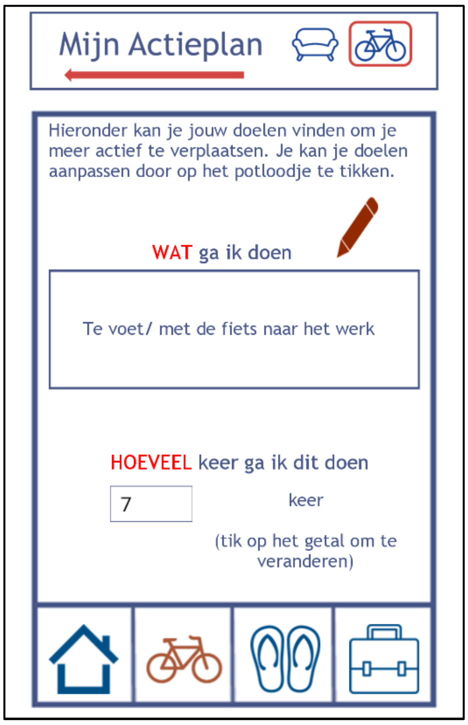

Translation:

"Below you can find your goals to do more active transport. You can adapt your goals by tapping on the pencil icon."

Figure 1b. Coping planning module: hindrances and solutions

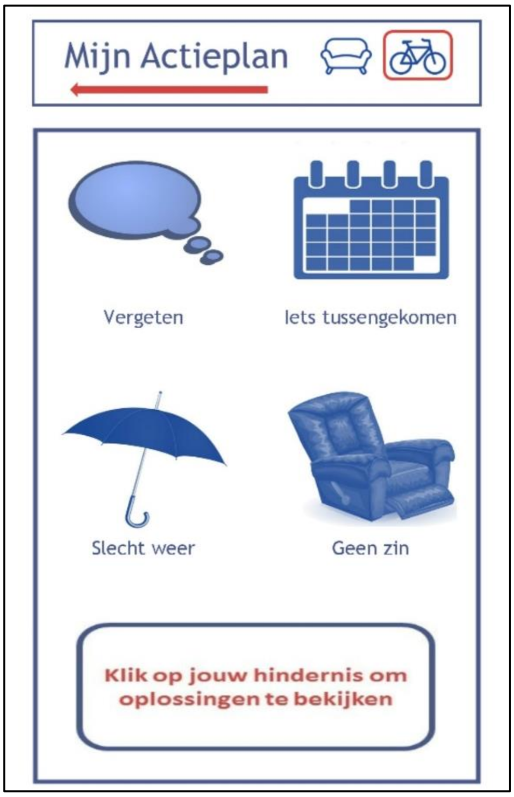

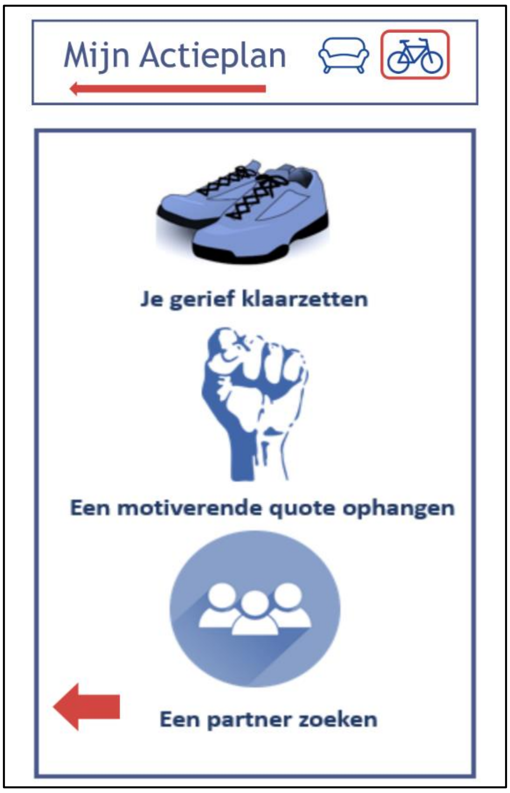

Text in red:
"Tap on a hindrance to find solutions"

Figure 1c. Monitoring module

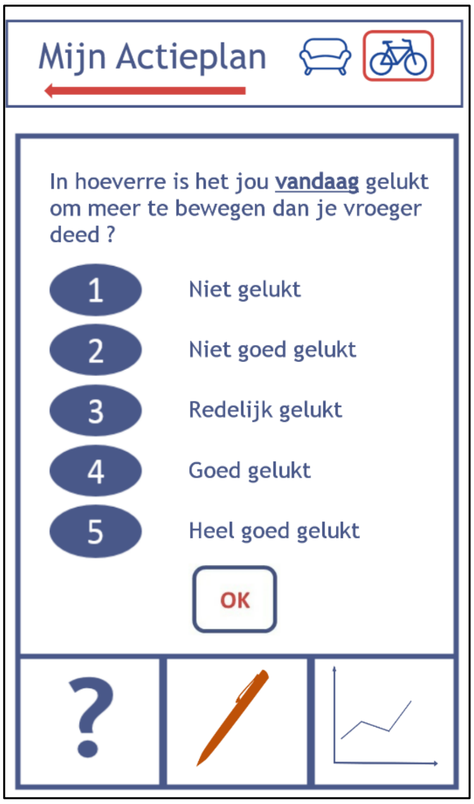

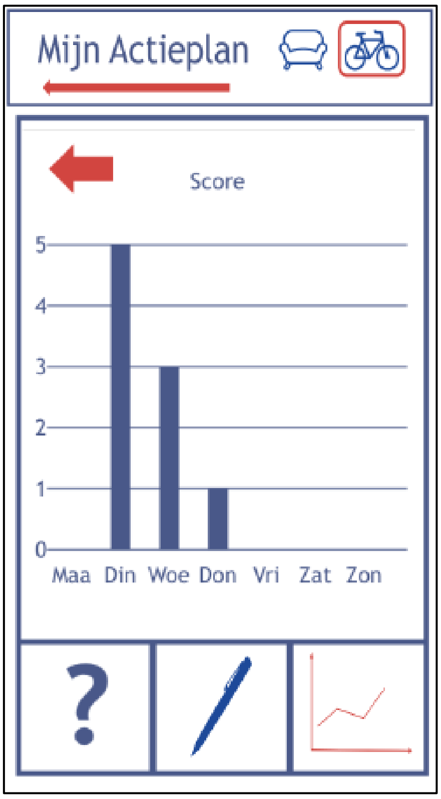

Translation:

“Today, to what extent did you move more than you did before?”

1= not succeeded

5= very well succeeded

Figure 1d. Quizzes

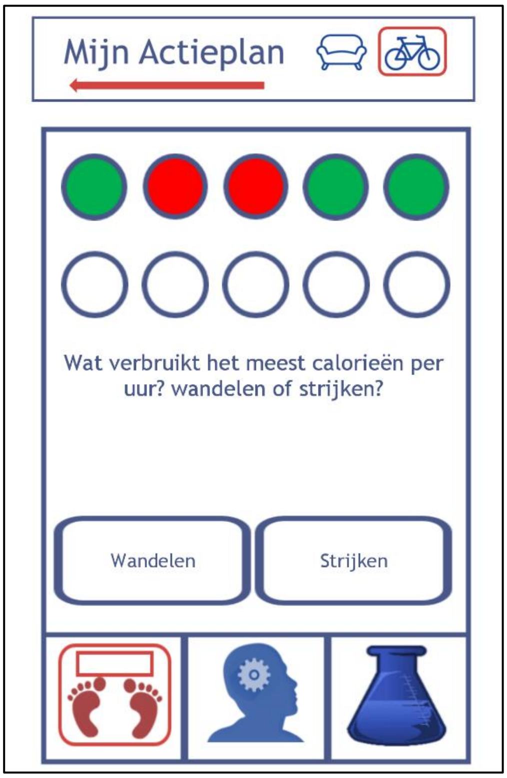

Translation:

“With which kind of movement you use the most calories? Walking or ironing?”
